# Upright and supine particle therapy of lung cancer: A 4D dosimetric comparison

**DOI:** 10.1002/mp.70377

**Published:** 2026-03-03

**Authors:** Maria Chiara Martire, Lennart Volz, Marco Durante, Mark Pankuch, Christian Graeff

**Affiliations:** ^1^ Biophysics GSI Helmholtzzentrum für Schwerionenforschung Darmstadt Germany; ^2^ Department of Electrical Engineering and Information Technology TU Darmstadt Darmstadt Germany; ^3^ Institute for Condensed Matter Physics TU Darmstadt Darmstadt Germany; ^4^ Northwestern Medicine Proton Center Warrenville Illinois USA

**Keywords:** carbon ion therapy, lung cancer, upright therapy

## Abstract

**Background:**

A renewed interest in upright particle therapy is currently driven by the availability of upright positioning and imaging systems. The upright positioning system could enhance fixed beamlines for effective carbon ion treatments in central body regions, with a substantial cost and space advantage. In addition, few studies have suggested advantages in patient breathing and lung volume in an upright posture. Comparative dosimetric analyses are needed to determine the clinical viability of upright patient positioning for carbon ion therapy of thoracic cancers but are challenged by various sources of bias.

**Purpose:**

To provide a comprehensive analysis of all parameters influencing the comparison between upright and supine carbon therapy of thoracic patients through 4D dosimetric studies.

**Methods:**

Paired upright and supine 4DCTs were available for six patients treated at the Northwestern Medicine Proton Centre (NMPC), under the Proton Collaborative Group (PCG) registry. Deformable image registration (DIR) between upright and supine CTs was performed on a region of interest (ROI) including the rib cage for target propagation, to avoid failure in DIR caused by thorax anatomical differences. DIR quality was evaluated on lung structures through Dice similarity coefficient (DSC) and average Hausdorff distance (AHD) metrics. Paired 3D plans were optimized on the originally contoured and propagated target volumes, to investigate the effect of segmentation differences. The impact of beam geometry choice was investigated by optimizing plans with a variety of treatment angles. Single‐fraction and accumulated 4D doses were calculated with the research treatment planning system TRiP4D to analyze the impact of differences in breathing‐induced tumor motion in the two postures. Plan quality between upright and supine plans were assessed through *D*_95%_, HI, and *V*_95%_ for the internal target volume (ITV) and V16Gy(lung) and V20Gy(heart) for lung and heart, respectively.

**Results:**

Restraining DIR on the ribcage ROI enabled successful DIR. Within the ribcage ROI an average AHD of 1.5 mm and DSC of 0.95 was achieved on the propagated lung structure. Position specific angle selection showed vertical posterior/anterior beams might not be optimal for upright treatments. Comparable 3D treatment quality was achieved for five patients, while an increase of 5 pp occurred in V20Gy(heart) and V16Gy(lung) of patient P6 in upright. The 4D study showed the different positions have clinically relevant impact, increasing *D*_95%_ of 3 pp for one patient with halved motion amplitude in upright posture. In addition, robustness was similar between postures, even with a more conservative 5%/5 mm uncertainty setting for upright. When assuming only a fixed beam line is available, as is the case for most carbon ion centers, a comparable plan quality with 360° beam angle flexibility in upright position was observed.

**Conclusions:**

The presented work comprehensively evaluates the influence of various parameters on the comparison of upright and supine therapy of thoracic patients. A solid understanding of these parameters is paramount to reduce bias in future larger patient cohort studies on the viability of upright positioning. The final dosimetric comparison between postures highly depends on patient characteristic and the investigated parameter. More data are needed to provide a resilient comparison between postures.

## 1 INTRODUCTION

The interest in particle therapy (PT) as an advanced form of radiotherapy has increased widely over the last decades.^1^ The dose deposition of charged particles, characterized by a low entrance dose and a sharp Bragg peak (BP), allows for a precise target coverage with enhanced normal tissue sparing compared to photon therapy. With particles heavier than protons, like carbon ions, a sharper depth dose deposition, a smaller lateral beam spread, and a higher biological effectiveness can be exploited.^2^ Thus, carbon ion radiation therapy (CIRT) has become a promising modality to treat radioresistant tumors, such as non‐small cell lung cancers (NSCLCs).^3^, ^4^ Nevertheless, physical and biological advantages of PT are penalized by higher technological hindrances, especially for heavy ions. A major investment for a CIRT facility is related to the massive and expensive gantry.^2^, ^5^ Gantry‐less treatment solutions, such as upright patient positioning in front of a horizontal beam nozzle, could make CIRT accessible on a larger scale, conditional on whether they enable treatment of similar quality. Upright patient positioning would facilitate the rotation of the patient in front of a fixed beamline for realizing different treatment angles. Indeed, as shown by Yan et al.,^6^ a 360° rotating chair allows for the clinically desired beam angle flexibility without the necessity of a gantry. In another study from Yan et al.,^7^ they reassessed the necessity of the gantry for proton treatments of various treatment sites, for the case a seated patient positioning option can be exploited, showing promising results. Upright positioning, by removing the need for a gantry, could combine physical and biological advantages of PT with a reduced facility cost, footprint, total shielding material, and single field treatment time.^8^, ^9^, ^10^ The renewed interest in upright therapy, based on the above‐mentioned arguments, was recently met by the development of upright imaging and patient positioning systems.^11^, ^12^

To justify the many changes necessary for upright PT, besides economic arguments, clinical impact studies verifying the quality of upright treatment plans are paramount. The altered relative gravity direction causes anatomical changes throughout the patient's body, especially in the abdomen and chest. Recent studies indicated an increased lung volume and a decreased breathing motion, which can be speculated to enable a lower lung mean dose and a decreased interplay effect magnitude, respectively.^13^, ^14^, ^15^, ^16^ However, the anatomical changes following the change of posture and their clinical impact are patient‐specific, and thorough treatment planning studies comparing supine and upright therapy under consideration of robustness and breathing motion are lacking.

When comparing different patient postures, multiple variables can affect the final dose distribution and may introduce bias. For example, the choice of beam angles for supine treatments can be made assuming gantry availability or fixed beamlines. For CIRT, currently only 4 gantries are available at the 16 total facilities.^1^ Therefore, constraining the angle choice to fixed beamline treatments for supine and chair treatments for upright better reflects the reality at the majority of facilities. Further, robustness evaluation requires understanding the patient setup reproducibility,^17^ where available margin recipes are designed to accommodate the setup reproducibility of recumbent patients and may require to be redefined for upright postures.^18^ Choosing the same setup uncertainties for upright postures may be inadequate, where current lack of clinical experience and of immobilization equipment that, like for supine, has been continuously optimized for decades of clinical application, may entail larger positioning errors. Further, because of a lack of magnetic resonance imaging (MRI) and positron emission tomography (PET) scans for upright positions, these imaging techniques are currently not available to support target delineation, which is instead solely performed on diagnostic computed tomography (CT) images.^11^, ^12^ This may introduce increased uncertainties on target delineation in an upright posture. In addition, for available paired CT data, patients eventually have been treated in either supine or upright position. If data is not recontoured specifically for dosimetric comparison studies, it is reasonable to assume the quality of delineations to be better for the posture actually chosen for treatment.

In this work, we rigorously analyze the impact of the above mentioned parameters with the aim of providing a recommendation for a resilient comparison framework between supine and upright CIRT of thoracic patients. First, a deformable image registration (DIR) method for target contour propagation between supine and upright CTs of the same patients is presented. Subsequently, a treatment planning study was conducted, and 4D dose distributions were calculated on supine as well as upright 4DCT data with the research treatment planning system (TPS) TRiP4D.^19^, ^20^, ^21^, ^22^ Motion related dose degradation, dose accumulation over multiple fractions, plan robustness, and the dosimetric impact of beam angle choice and flexibility were investigated.

## 2 MATERIALS AND METHODS

**FIGURE 1:**
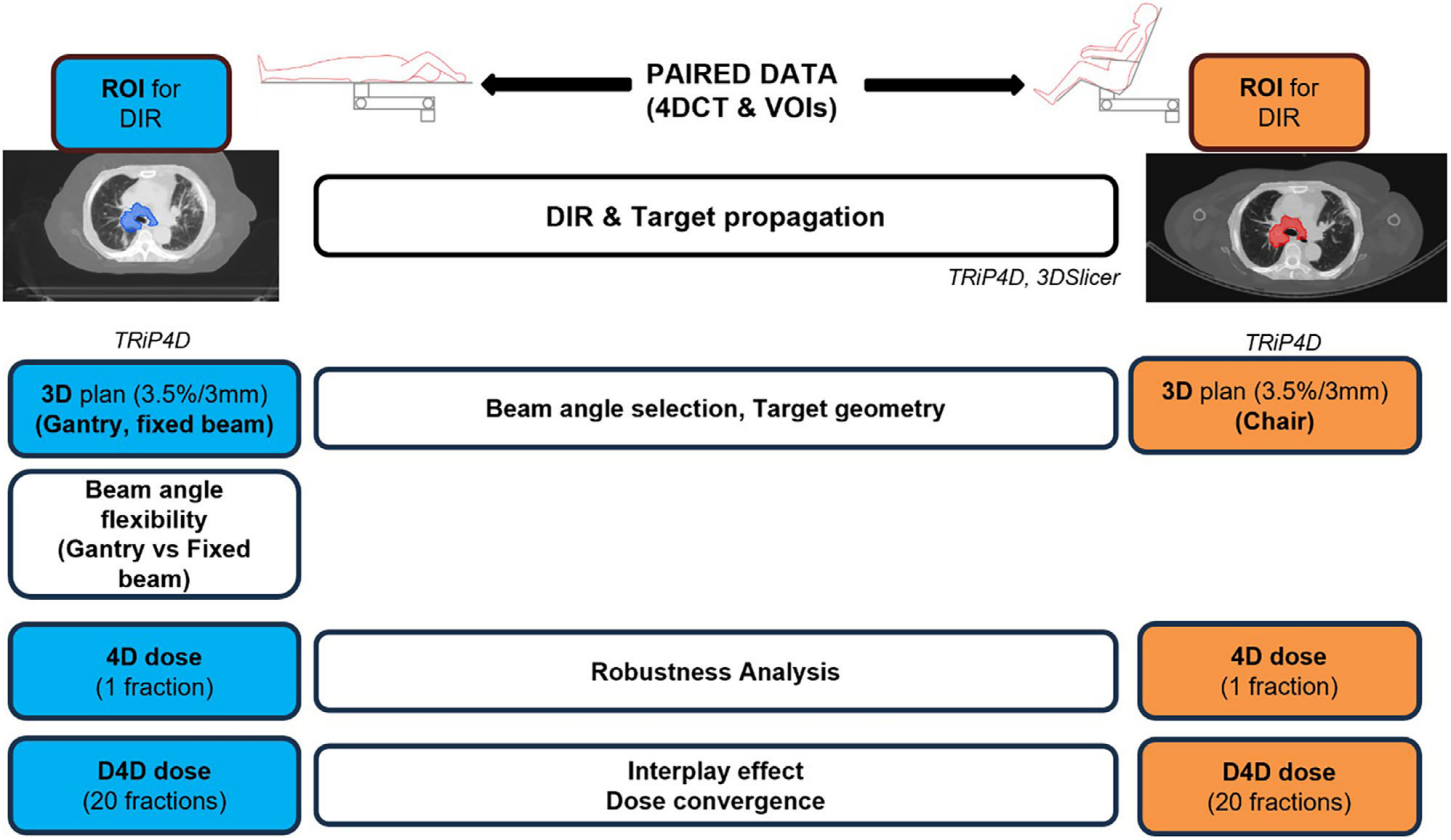
Upright and supine image registration and treatment plan comparison method workflow. D4D dose, dynamic 4D dose/interplay dose; DIR, deformable image registration; Opt., optimization; ROI, region of interest; VOI, volume of interest.

Figure 1 shows the workflow to identify the impact of various parameters for a resilient comparison between postures. First, the impact of target volume differences is identified and a DIR method for target propagation is developed, to eliminate possible bias from different contouring quality and substitute missing contours in either posture. Then, a 3D study was performed to assess the impact of beam angle selection and target geometry in both positions. 4D doses were calculated considering (dynamic 4D (D4D] doses) and neglecting (4D doses) the interplay effect to investigate both motion related uncertainties and plan robustness in the two positions.^23^ D4D doses were also employed to assess the effect of a fractionated treatment on the dose distribution.

### 2.1 Patient data

Paired 4DCTs data of six thoracic cancer patients, treated under the Proton Collaborative Group (PCG) registry, were available from the Northwestern Medicine Proton Center (NMPC) in Warrenville, IL, USA. Patients P1 and P2 were treated for reirradiation. For each patient, a 4DCT was acquired both in supine (650 mm FOV, 2.5 mm slice distance, 1 mm resolution, and 10 motion phases) and upright (600 mm FOV, 3 mm slice distance, 1 mm resolution, and 10 motion phases) positions. In supine, patients were positioned with the arms up. In upright, patients were seated in a chair with a 20° backrest incline with their arms down on an arm rest. The patient characteristics of interest are listed in Table 1 for both immobilization postures. Organs at risk (OARs), that is, both lungs and heart volumes, and target contours, that is, the geometrical internal target volume (ITV), were available in both positions for all patients, except for patient P2 and P4 for whom the ITV was only contoured on the upright and supine images by the NMPC clinical staff, respectively. Contoured ITVs are listed for both supine and upright positions, together with the propagated ITV in upright. These values are relevant for the study since the nominal upright 3D plans were optimized on the propagated structures. For completeness, it is important to specify that patients P1, P3, P4, and P5 were treated at the NMPC in supine position, whereas patients P2 and P6 in upright. In addition, the tumor displacement, calculated as the average magnitude of the deformable vector field (DVF) transforming the target voxels from the end‐inhale (0% respiratory cycle) to the end‐exhale (50% respiratory cycle) phase, is listed as Δmotion in Table 1 for each patient.

**TABLE 1:** Patient characteristics for the upright and supine position.

		Target volume (cc)			Target Δmotion (mm)		Lung volume (cc)		Heart volume (cc)	
Patient	Target position	Supine (Cont.)	Upright (Cont.)	Upright (Prop.)	Supine	Upright	Supine	Upright	Supine	Upright
P1	RUL	60	65	58	2	3	2259	2360	858	782
P2	LLL	–	20	–	6	3	3921	4586	706	633
P3	Mediastinum	381	340	364	3	2	3381	3957	497	590
P4	LUL	60	–	56	5	4	3251	3473	794	915
P5	LUL	273	244	262	2	2	2644	3166	717	842
P6	Mediastinum	223	195	249	5	6	3247	3109	695	722

The volumes of the target, heart, and lungs are listed. Upright target volumes originally contoured (Cont.) and propagated (Prop.) from supine geometry are shown. In addition, the target position in the lung is indicated. The target motion, calculated as the average magnitude of the deformable vector field transforming the target voxels from end‐inhale to end‐exhale phase in the 4DCT, is provided as Δmotion. For patients P2 and P4, the target was only contoured in upright and supine position, respectively. LLL, left lower lobe; LUL, left upper lobe; RUL, right upper lobe.

### 2.2 Treatment planning and robustness analysis

Treatment planning in this work was conducted using the treatment planning software TRiP4D. TRiP4D features state‐of‐the‐art infrastructure for 4D dose computation, such as robust optimization considering the variable biological effectiveness of carbon ions, 4D optimization, and computation of D4D doses to quantify the interplay effect.^23^ For estimating the carbon ions’ relative biological effectiveness (RBE) we used the local effect model (LEM I) with a global alpha/beta ratio of 2. All plans were 3D‐optimized on the reference CT for a dose of 3 GyRBE/fraction to the geometrical ITV after density override (70 HU), only coplanar beams were considered, and voxel‐wise robust optimization with 3.5% range and 3 mm setup uncertainty was applied.

The plan quality was evaluated through dose distributions and dose volume histograms (DVHs), visualized in 3D Slicer.^24^ DVH metrics of interest comprised *D*_95%_ (prescribed dose percentage delivered to 95% of the target volume), *V*_95%_ (target volume percentage receiving at least 95% of the prescribed dose) and homogeneity index (HI = *D*_5%_–*D*_95%_) to quantify the target coverage, and V20Gy(heart) and V16Gy(lung) were used as the reference metrics for heart and lung, respectively. The OARs metrics derive from V20Gy(lung) and V25Gy(heart) used in the clinic for the standard 2 GyRBE/fraction in 30 fractions treatment and converted to the corresponding values for 3 GyRBE/fraction in 20 fractions used in this study. The plan was considered clinically acceptable for *D*_95%_ and *V*_95_% greater than 95%, and an HI lower than 5%, as well as heart V20Gy(heart) lower than 10%, and V16Gy(lung) less than 30% for the whole lung.^25^ The target coverage was prioritized over lung and heart doses. The unfavorable tumor position and dimension of some patients prevented optimal lung and heart sparing. Further specific planning details for each step of the comparison framework are provided in the next sections.

A robustness analysis (RA) was performed on nine scenarios (nominal scenario, 3 mm translations in each cardinal direction, and 3.5% undershoot/overshoot scenarios). The scenarios were calculated from the 4D doses, namely the weighted average of the doses calculated from the individual motion phases and cumulated on the reference one, neglecting the correlation with beam delivery timing.^26^

Current margin recipes and robust optimization settings for range and setup uncertainties are derived from experience and population‐based analyses.^18^ These may not apply to an upright position, due to an increased anatomical uncertainty for the upright posture at least for early adopters of new upright technology, and possible sagging during treatment.^27^ We therefore expanded the RA of both positions to more conservative 5% range and 5 mm position uncertainties.

### 2.3 Deformable image registration and target propagation

Upright to supine DIR is challenged by the considerable differences in patient anatomy that come with changing the posture. Due to the shortage of MRI in the upright posture as well as limited time of radiologists, it is reasonable to assume that the quality of the target contour differs between upright and supine positions. For example, in two of the patient cases, the target was contoured only in the position in which the patient was eventually treated in. Changes in target delineation quality is a source of uncertainty in a dosimetric comparison, for example, due to differences in the target volume, therefore it is important to understand and quantify it. We performed target contour propagation between postures for achieving comparable target volumes in both postures.

Conventional DIR methods^24^ fail to correctly register the two postures due to the vastly different anatomy, such as arms‐up versus arms‐down positioning, patient sagging with different spine curvature, and displacement of adipose tissue. We constrained DIR to a ribcage region of interest (ROI), masking the CT in the reference breathing phase (end‐inhale phase) for each supine and upright 4DCT. Because of the lack of a target ground truth, DIR quality analysis (QA) was performed inside the ROI using the lung VOI. Dice similarity coefficient (DSC) and average Hausdorff distance (AHD) metrics were calculated between contoured and propagated lung VOI to assess overlap accuracy and spatial agreement.^29^ DSC values above 0.9 are considered excellent, while AHD values should be smaller than the CT slice distance.^30^ Target propagation QA was performed comparing ITV shape, volume, and center of mass (COM) distance within the thorax. The ribcage ROI definition is described in detail in Supporting Information S1, together with registration and target propagation QA.

After DIR and target propagation between paired CTs, the ITV was available in both positions for each patient. In particular, the contoured ITV was used as planning target in supine for all patients except for P2, for which the propagated ITV from the upright CT had to be used. For the upright posture, the propagated ITV was used as planning target for all patients, except for P2, for which the contoured ITV was used. The impact of a different target geometry and the beam angle selection was accounted for in the 3D study, as described in the next section.

### 2.4 Choice of beam angles

When considering comparative treatment planning studies between immobilization postures, an important source of possible bias is the choice of beam geometry. To understand its impact and eventually propose a suitable beam geometry for thoracic treatments in upright position, we followed the following main steps:

- Optimize a nominal supine plan following the general clinical standard for thoracic patients (360° flexibility is assumed)
- Use the same optimized “supine” beam directions for upright (360° flexibility is assumed)
- If necessary, optimize beam selection in upright to achieve a non‐inferior plan (360° flexibility is assumed)
- Compare the original supine plans with fixed beam geometry plans as available only in most CIRT facilities

The second step allowed to understand if the clinical standard beam selection in supine can be directly translated to the upright case. We used two fields for each patient following the protocol at the NMPC and assumed gantry‐availability in supine to make the comparison more robust. For consistency, beam angles are reported in the patient coordinate system, since otherwise two different coordinate systems would be used for the gantry and the chair. All patients, except P1, have the tumor located posteriorly to the heart, which suggested, for most cases, the use of posterior–anterior (PA) or posterior‐oblique beam directions. In addition, to reduce the beam path inside the lung, vertical beams are preferred to oblique ones, and extreme beam inclinations (typically more than 45°) are not recommended because of the shoulder/arms intersecting the beam path, which typically increases range uncertainty.

If the upright plan was inferior to the supine one, a better configuration was investigated for this position, aiming to an at least comparable plan. With this step, the optimal beam direction for the upright position was found, and the 3D nominal plan was defined. The optimization of these plans was performed on the propagated upright ITV to neglect the target geometry differences. For completeness, the same optimal angles were used to optimize 3D upright plans on the original contoured ITV, where available.

Following this beam selection strategy, supine and upright nominal plans were compared in terms of the previously mentioned heart and lung metrics, as target coverage was similar in all cases.

Since currently 12 out of 16 CIRT facilities do not have a gantry, assuming full 360° flexibility for supine is not representative for the majority of CIRT rooms. To quantify the impact of this on the plan comparison, we also optimized the supine plans for fixed beamlines, including a vertical, horizontal, and a 45° inclined beam, as available at SAGA‐HIMAT center in Tosu, Japan. The other plan optimization parameters were kept the same as for all other investigations. In this step, dose calculation was performed neglecting motion to avoid additional complexity.

### 2.5 4D delivered dose analysis

Finally, the plan comparison between postures should be performed on D4D doses, that is, considering both the pencil beam scanning and patient breathing motion,^26^ to acknowledge any differences in interplay‐effect related dose degradation between the two treatment positions. For this purpose, plan delivery was simulated using an in‐house beam delivery simulator,^19^ assuming the synchrotron accelerator and beamline characteristics at Heidelberg Ion Beam Therapy Center, Marburg Ion Beam Therapy Center, and Shanghai Proton and Heavy Ion Therapy Center. This includes dynamic beam intensity control^31^ taking into account minimum spot‐dwell times, and realistic handling of spills and spill‐pauses with variable spill lengths of up to 4 s, and spill pauses of 4 s.

The interplay effect strongly depends on both the motion surrogate and the breathing phase at the start of the treatment. Since the real motion patterns for these patients were not available, we used the breathing trajectory description described by Lujan et al.^32^ as a motion surrogate, assuming a fixed breathing period of either 5 s or 10 s. Per patient 20 dose calculations were performed, that is, one dose calculation per each of the 10 starting phase for either breathing period. We chose the same surrogate function for both postures, it is important to note that since the anatomical motion is contained in the 4DCT, this only affects the breathing period. Upright and supine interplay dose distribution differences were assessed with a Wilcoxon signed Rank test, considering *p* < 0.05 as statistically significant.

Aside from single fraction doses, we also simulated a full treatment course with 20 fractions. From the pool of 20 dose calculations, four treatment courses were generated, random sampling 20 individual fractions from the available data, and summing them up to obtain a total cumulative dose of 60 GyRBE. Aside from analyzing the total doses, the obtained fraction‐wise cumulative doses were compared against the cumulative planned dose. This reveals the convergence of the interplay‐effect doses toward the ideal‐rescanned dose as a function of the number of fractions in terms of target coverage (*D*_95%_ and HI). The convergence speed is defined as the number of fractions necessary to achieve the metric value plateau (±1%), which ideally is above the minimum clinically acceptable limit for the specific metric (i.e., 95% for *D*_95%_).

## 3 RESULTS

### 3.1 Anatomical differences

Because of the significant anatomical differences occurring between the recumbent and the upright position, dose distributions can change considerably when a different treatment posture is chosen. Particularly, the heart and lung volumes are structures of interest for thoracic patients. From the manually contoured lung VOIs, for patients P1 to P5, an average increase of 17.4% ± 9.9% and 10.3% ± 6.4% in volume was found for the upright left and right lung, respectively, giving an average increase of 13% ± 6.1% for the total lung volume in upright posture, as shown in Table 1. Patient P6 showed a 4.4% lung volume decrease in upright position. An average increase of 6% was also found for the upright heart VOIs. The lung volume variations can be addressed both to the contouring output precision and to a different lung capacity in the upright position. Meanwhile, heart volume variations mostly derive from the difference in VOI contouring quality. Supine and upright target motions are listed for each patient in Table 1, showing an average motion amplitude difference of 1 mm. Only for patient P2, the target displacement decreases from 6 mm (supine) to 3 mm (upright).

**FIGURE 2:**
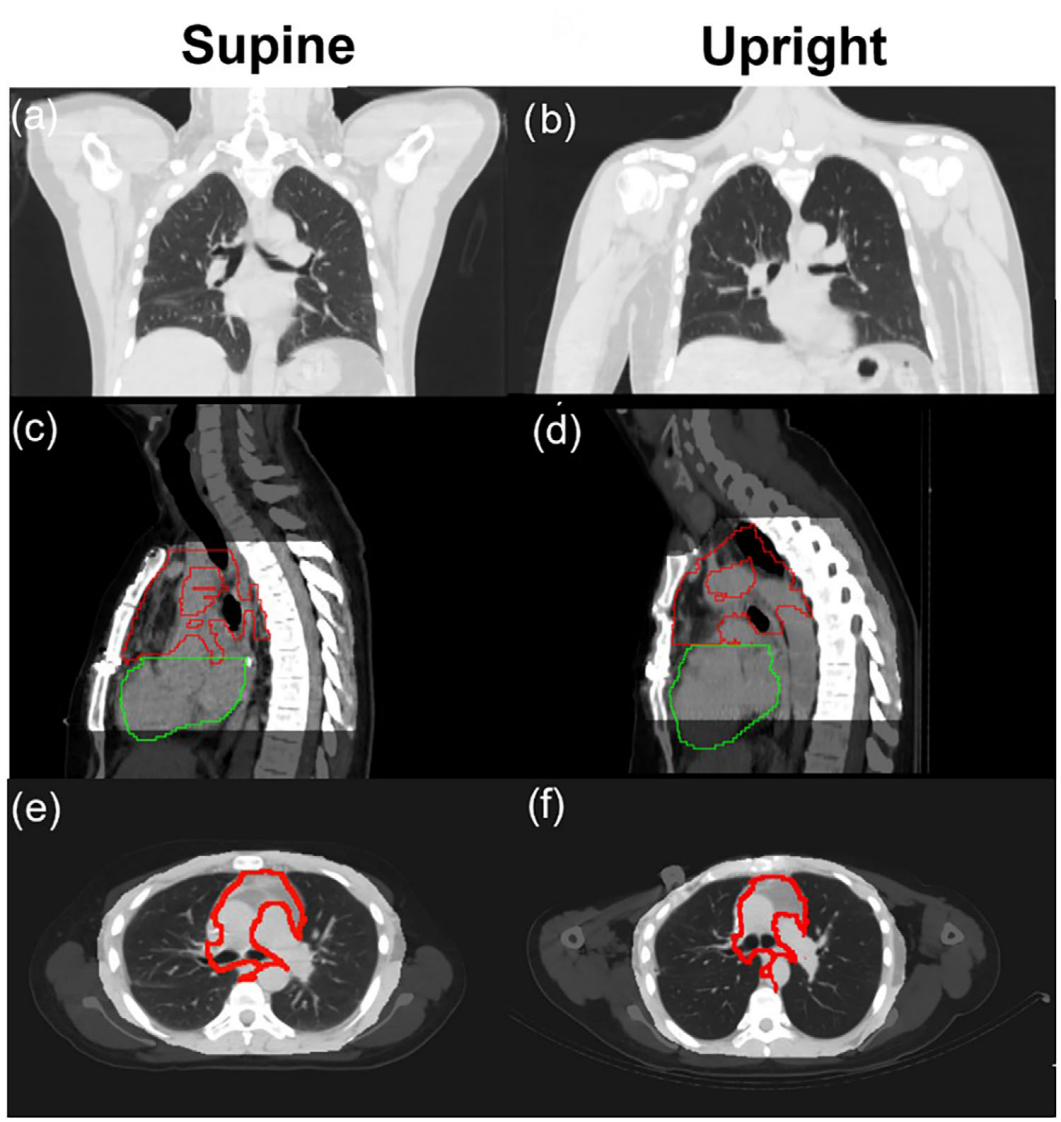
Patient P3 supine (a, c, e) and upright (b, d, f) reference CT in coronal (a, b), sagittal (c, d) and axial (e, f) view. Body position (a, b) and target (red) and heart (green) contour (c, d) differences are highlighted. The ribcage region of interest (ROI) is shown in sagittal (c, d) and axial (e, f) view superimposed to the original CT. The original (e) and propagated (f) target are also shown.

In Figure 2, upright and supine anatomical differences are shown for patient P3, including different positions of arms, shape, size, and position of contoured ITV and heart and the matching shape between manually contoured and propagated ITV. Notably, the heart VOI is artificially cut at the border with the target VOI, in both volumes, same as the ITV contour at the edge of the heart on the upright CT.

### 3.2 Image registration and target propagation

Registration and target propagation QA as well as qualitative and quantitative target registration results are shown in Supporting Information S1. A brief summary is given here. DIR and propagation QA showed good agreement between propagated and contoured structures. Lung VOI comparison yielded an average DSC of 0.95 and AHD of 1.5 mm, indicating accurate registration. Across all patients and both propagation directions, ITV volumes differed by 5.7% ± 3.8% on average. COM distances were generally within a few voxels, with the largest discrepancy observed in patient P1.

### 3.3 Treatment plan comparison

#### 3.3.1 Choice of beam angle

Following the method described in Section 2.4, for each patient, we optimized a supine 3D plan, based on clinical guidelines for thoracic patients. Table 2 shows DVH metrics comparison for heart and lungs, since the same target coverage (*D*_95% _= 98%, HI = 1.8%, and *V*_95% _= 100%) was achieved for all plans. Applying the same angle selection as for the supine plans (1st and 2nd column) resulted for the upright posture in increased OAR doses (3rd column), in all patients except P1, suggesting that a new angle optimization is necessary. The beam angles optimized for the upright geometry are shown in the fourth column and applied both to the propagated (5th column) and contoured (6th column) target in upright.

**TABLE 2:** Patients P1–P6 supine (2nd, 3rd columns) and upright (5th, 6th columns) 3D plans.

		Supine (Angles opt., Con. Target)		Upright (“Supine” angles, Prop. Target)			Upright (Angles opt., Prop. Target)		Upright (Angles opt., Con. Target)	
Patient	“Supine” angles (°)	V16Gy (%)	V20Gy (%)	V16Gy (%)	V20Gy (%)	“Upright” angles (°)	V16Gy (%)	V20Gy (%)	V16Gy (%)	V20Gy (%)
P1	90,290	10.4	0	9.9	0	90,290	9.9	0	10.4	0
P2	250,290	9.4^a^	5.5^a^	11.0^a^	8.6^a^	250,335	–	–	11.3^a^	7.8^a^
P3	90,270	23.0	15.0	23.5	18.6	90,270	23.5	18.6	20.1	7.6
P4	270,290	4.3	2.8	4.7	4.4	270,290	4.7	4.4	–	–
P5	270,135	17.3	8.4	21.9	6.0	225,135	18.2	6.8	16.9	5.7
P6	250,290	19.9	5.0	24.6	9.4	250,290	24.6	9.4	24.1	7.4

For each plan V16Gy(lung) and V20Gy(heart) are listed. In 1st to 3rd columns, the angles optimized for supine (“Supine” angles) and resulting plans on supine contoured target (Con. Target) and upright propagated target (Prop. Target) are shown. In 4th to 6th columns, the angles optimized on upright (“Upright” angles) and resulting plans on upright propagated target and contoured target are shown.

^a^ Patient P2 supine plans optimized on propagated ITV and upright plans optimized on contoured ITV. Vertical AP 90°, vertical PA 270°, oblique AP 45 and 135°, oblique PA 225 and 315°. Field angles refer to the patient coordinate system and not the room system that would be different for the gantry and the chair.

For patient P2, an upright specific angle selection (second field inclined from 20° PA to 65° PA) allowed to achieve a lower V20Gy(heart) but the V16Gy(lung) remained elevated compared to the supine plan. Nevertheless, the V16Gy(lung) was still below the clinical acceptance threshold.

**FIGURE 3:**
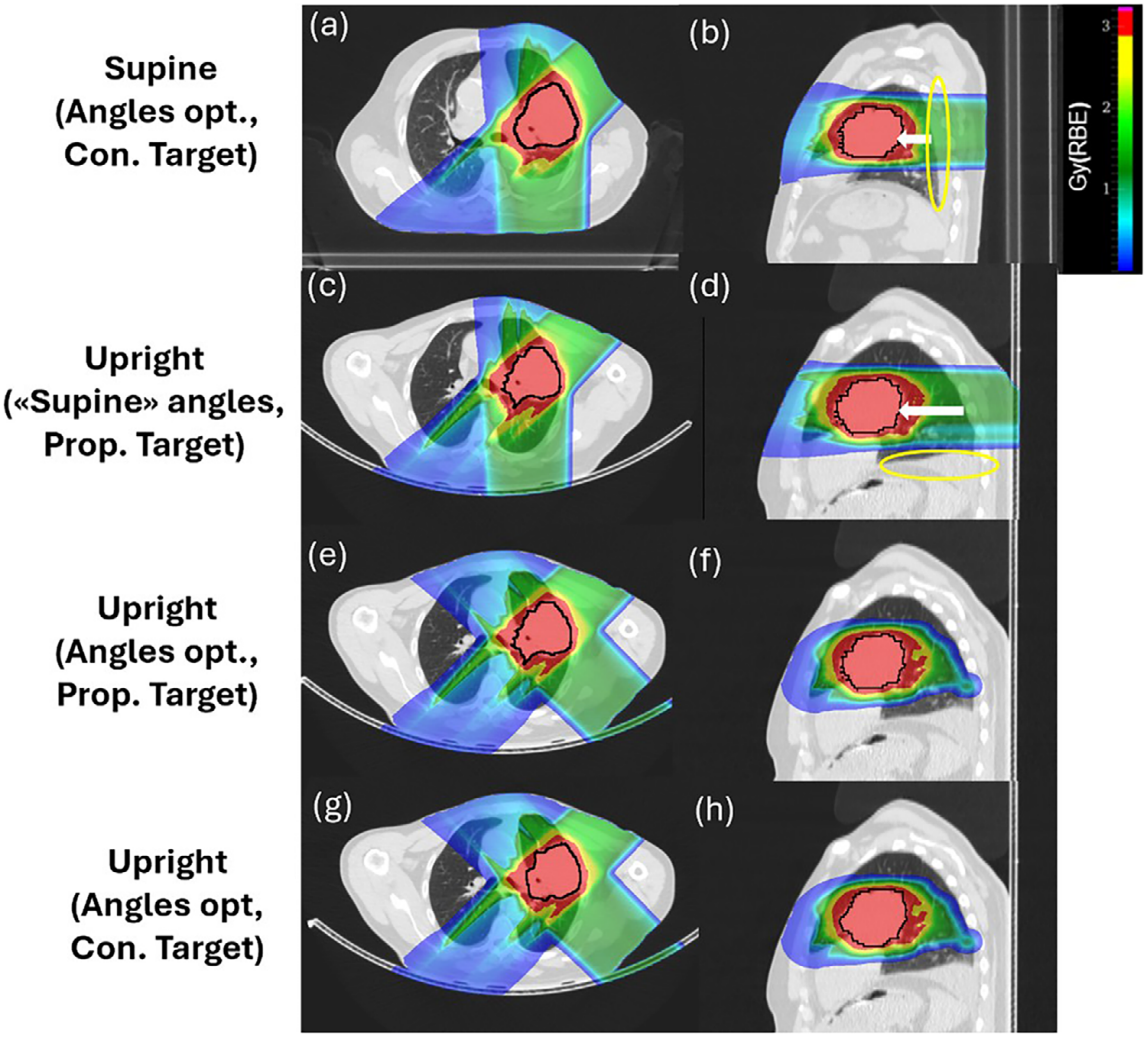
Patient P5 supine (a, b) and upright (c–h) 3D plan dose cuts in axial (a, c, e, g) and sagittal (b, d, f, h) view. Supine nominal plans are shown, after beam angle optimization (Angles opt., Con. Target) (a, b). The same angles are chosen for the upright plan (“Supine” angles, Prop. Target) (c, d). The changed position of the pleural effusion posterior (b) or inferior (d) is marked in yellow. This results in a longer posterior beam path through the lung in upright position, and an increase in V16Gy(lung), see Table 2. Upright plans after beam angle optimization on the upright geometry (Angles opt.), optimized on the propagated (Prop. Traget) (e, f) and originally contoured ITV (Con. Target) (g, h) are shown. The target is shown in black. Posterior lung volume changes are marked with white arrows. Con., contoured; Opt., optimization; Prop., propagated.

For patient P5, a new angle in upright permitted to reduce both V16Gy(lung) and V20Gy(heart) to values comparable to the supine position. As shown in Figure 3, in the upright posture the lung is compressed less in the PA direction compared to the supine position. This results in a longer beam path in the lung for PA fields. Therefore, the oblique field direction allowed to reach a reduced V16Gy(lung) with respect to the vertical beam that was the optimal choice in supine. As a result, for this patient, the optimal field was inclined to 45° compared to the initial PA direction to achieve reduced OAR doses.

For P3, P4, and P6, the nominal supine angles (opposing vertical AP/PA fields for P3 and P4, and PA fields with ± 20° inclination from the vertical axis for P6) also resulted in the best plans for the upright posture. An increased V20Gy(heart) was found in upright for P3 and P4, as well as V20Gy(heart) and V16Gy(lung) in upright for P6, independently on the beam angle selection. For P3, this was due to the target surrounding the heart, which made proper sparing of the heart impossible. For P6 and P4, because of patient‐specific lung anatomy, the upright plan always resulted in higher OARs dose, independently on the beam angle configuration.

The plans described in this paragraph, using upright‐specific angles optimized on the propagated target, are shown in the fifth column of Table 2; these plans are the reference upright plans for the remainder of the study. The impact of the target propagation is seen when the optimal beam angles are kept but the upright plan is optimized on the original target contour instead of the propagated one (6th column). For patient P3 the V16Gy(lung) and V20Gy(heart) showed a higher difference with the propagated target plan. Specifically, an increase of 3 pp and 11 pp was found for lung and heart doses, respectively. This is in accordance with Figure 2, where the target contour quality in the two positions is visibly different. In particular, the upright target VOI was artificially modified to avoid heart overlap. As mentioned, the dataset of patients P2 and P4 did not include an originally contoured ITV in both positions, therefore no comparison was possible.

As described in Section 2.4, an additional comparison between gantry and fixed beam plans for supine geometry is necessary to quantify the dosimetric advantage of a full 360° angle flexibility. Each supine nominal plan (2nd column of Table 2) was re‐optimized, only considering vertical, horizontal and/or 45° beams. The results, listed in Table S4 of Supporting Information S2, show that for patient P3 and P5 the nominal optimal 3D plan would have also been deliverable with fixed beams. For patient P1, the plan quality was not affected by a fixed beam availability, while for patients P2, P4, and P6 increased dose was delivered to the OARs because of the limited possible angles selection, in particular, an increase of 3 pp, 3 pp, and 9 pp for P2, P4, and P6 V16Gy(lung) was found. When only a fixed beamline is available, the upright position offers increased benefit for these patients, compared to the gantry‐based comparison.

#### 3.3.2 Robustness analysis

In all patients, all scenarios delivered acceptable target dose for 3.5%/3 mm parameters, with *D*_95%_ and *V*_95%_ above 95% and HI below 5%, with no differences between treatment positions. The 5%/5 mm RA results are shown in Figure S2 of Supporting Information S3. In all patients and scenarios, in total only four scenarios did not pass the target to coverage criterium of *D*_95%_/*V*_95%_ > 95%. This occurs in patient P1 (1 scenario upright), patient P2 (1 upright, 1 supine), and patient P4 (1 supine).

#### 3.3.3 4D delivered dose analysis

**FIGURE 4:**
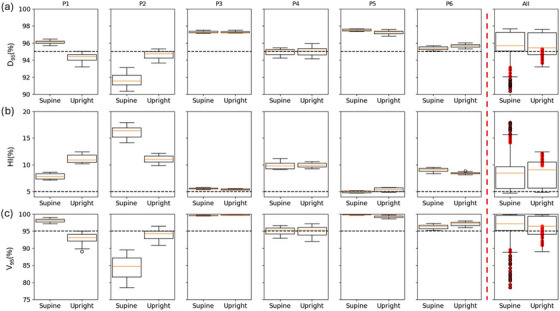
Upright and supine *D*_95%_, HI, and *V*_95%_ distributions over 20 deliveries. The first six columns show single patients results, in the last column, the averaged metrics distributions from the six patients are compared. Values deriving from patient P2 are plotted in red for each metric. Black dashed lines indicate the 95% and 5% threshold lines for *D*_95%_/*V*_95%_ and HI, respectively. Averages and standard deviations values for each metric are listed in Table S4.

For both upright and supine, 20 D4D dose distributions, that is, interplay‐considering dose calculations, were compared in target, lung, and heart to determine the effect of a different treatment position on motion‐related dose degradation. In Figure 4, upright and supine *D*_95%_ (a), HI (b), and *V*_95%_ (c) distributions are displayed for each patient. In Table S1 in Supporting Information S4, for each patient and metric, the average values and standard deviations are listed.

Statistically significant differences were found for patients P1, P2, P5, and P6. However, only for patients P1 and P2 the plan differences reached a clinically relevant magnitude, in favor of the supine and upright plan, respectively. For patient P2, a large dose degradation occurs in supine because of the 6 mm motion, and the plan quality was partially recovered in upright because of the substantial motion amplitude decrease. For all other patients, as shown in Table 1, the motion amplitude is not widely affected by the treatment posture, explaining the lower differences in the observed interplay effect.

Results for all patients are summarized in the last column of Figure 4 as well as in Table S5 in Supporting Information S4. No statistically significant differences were found between supine and upright plans for these patients. Values deriving from patient P2 are marked in red in the boxplot to further highlight the significant difference between treatment positions for this patient.

Since the interplay effect does not impact the dose distribution in normal tissues as much as in the target, for every patient, V16Gy(lung) and V20Gy(heart) values from the 20 deliveries were almost identical and very narrowly distributed around the average. The statistical analysis was therefore conducted with average values only. All OAR doses are reported in Table S6 of Supporting Information S4. For the lung, the results are consistent with what found in static plans, while for the heart the results are more diverse between patients.

The results of the comparison of plan convergence over the treatment course are shown in Figure S3 of Supporting Information S4. Consistent with the observations for single deliveries, only patient P1 and P2 show differences between postures, for P1 in favour of the supine and for P2 in favour of the upright plan. These differences are not statistically significant and almost disappear at the last fraction. For each patient, the dosimetric quantities converge after the same number of fractions in both postures.

## 4 DISCUSSION

We developed a framework to assess the impact of upright patient positioning on carbon ion therapy for thoracic patients by comparing plans generated for upright and supine therapy in different aspects. The work is based on upright and supine image registration and target propagation, as necessary to optimize the plan on the same target geometry and achieve a robust comparison. The dataset comprised paired 4DCTs of six patients treated at the NMPC under the PCG registry. For each patient, starting from the definition of an optimal supine 3D plan, multiple variables were considered and their impact on the dosimetric comparison assessed: anatomy changes and target geometry, beam angle choice and flexibility, plan robustness and convergence, and interplay effect magnitude.

### 4.1 Anatomy changes and target geometry

Lung volume increase, motion amplitude reduction, and heart volume changes are the anatomical variations of interest for thoracic patients^14^, ^15^, ^16^ and were also found in this study cohort. Based on a patient cohort of 15 patients, including the six patients used in the presented work, Marano et al.^15^ investigated thoracic anatomical changes between the upright and the supine body posture, focusing on heart and lung differences as structure of interest for therapy purposes. They reported a general increase in lung volume of 17% for left and 9.9% for the right lobe, consistent with what described in Section 3.1 of this work. Structure propagation is heavily affected by these anatomical changes if thoracic CTs of the same patient, acquired in two different body positions, have to be registered. For treatment planning study purposes, however, it is important that the target structure is propagated between postures, in order to disentangle effects associated with the change in posture from those that result from differences in the contouring. The construction of a ribcage ROI permitted successful target contour propagation.

In treatment planning studies, like the one presented in this work, multiple causes can be responsible for the differences between supine and upright plans. Choosing the parameter settings for comparison can introduce major bias in the final conclusions. Therefore, single effects from various sources need to be isolated and quantified. For example, if the geometry of the tumor differs between postures (either due to contouring differences or differences in the actual structure), this may change the dose to the surrounding critical organs and outweigh the effects from increased lung volume or reduced breathing motion. It is therefore important to understand whether observed effects reflect tumor morphology changes associated with the change in posture of whether the observed differences stem from differences in target contouring. It is important to be aware of the target contour as a source of uncertainty, as it can have a substantial effect on the conclusions drawn from a comparison study. If a perfect DIR was available, target contour propagation would eliminate this source of bias. However, DIR introduced uncertainties itself. As an observation from this work, we would therefore recommend to always perform the dosimetric comparison both for propagated and delineated target contours, to be able to quantify the difference between both approaches.

Here, we optimized upright carbon ion plans both on the contoured and propagated ITVs. A clear difference between postures was found for patient P3, for which the dose to the heart decreases by 11 pp when using the originally contoured ITV, compared to the propagated contour. A seemingly strong advantage in cardiac sparing in upright actually turned into a slight disadvantage when using comparable contours. In addition, for patients P5 and P6, the plan on the propagated target resulted in increased OAR doses. This illustrates the challenge of a fair dosimetric comparison between the postures with associated drastically changed anatomies. With the image registration and target propagation step, it was possible to isolate this variable.

Precise target and OARs contouring on upright CTs, therefore, remains a pitfall for treatment planning purposes. Because of the lack of clinical available MRI and PET upright imaging systems for radiotherapy, the VOI definition can only rely on diagnostic CT scans, characterized by a low soft tissue contrast. Schreuder et al.^33^ investigated two methods, based on synthetic CT or MRI images, to overcome this problem. To generate a synthetic image and perform intra‐modality registration, multiple steps are needed. In our case, the only required step preliminary to the registration is the definition of a ribcage ROI in both CTs. However, Schreuder et al. also suggested in their work the use of a restricted region as a possible approach to mitigate upright and supine anatomical differences.

### 4.2 Beam angle choice and flexibility

After a proper target propagation, it was possible to proceed with the plan optimization and dose calculations. The developed framework aims to investigate to which extent the standard supine plan optimization paradigm can be translated to the upright treatment modality. Therefore, we first optimized a nominal 3D plan for supine geometry, following the general standard for this treatment posture. When the same beam angle choice was translated directly to the upright CTs, it was possible to highlight the need of patient‐specific re‐evaluation of the clinical beam geometry.

In particular, as shown from patient P5 results (Figure 3), the ribcage smaller lateral extension and a less compressed lung in the seated position, implies a plan quality improvement in terms of dose to the lung when an oblique field is selected instead of a PA field. This patient highlights a caveat especially for lung cancer patients, where mobile effluence can significantly change the lung anatomy. While this resulted in a V16Gy(lung) advantage for the supine position, the effluence could also pose a significant risk for range uncertainty over the treatment course, while it was not in the beam path in the upright position. This suggests that the PA beam might not be the optimal treatment choice in upright position.

However, a limitation to the selection of oblique fields is the position of the arms/shoulders which would add substantial position uncertainties if placed in the beam path. Therefore, an upright position with arms up as implemented in some chair realizations,^8^, ^34^ could be advantageous. It is important to underline that these findings are patient‐specific. For example, for patient P6 an additional beam inclination did not allow for a reduction of the V16Gy(lung) in upright position. In particular, because of the more superior position of the heart, a significant increase in heart dose was observed. Marano et al.,^15^ using the same dataset of this work, also investigated the upright heart position changes in caudal direction, which might lead to smaller heart toxicity when the thorax is irradiated. However, consistently with the results from Chayt Marcus et al.,^35^ they found the inter‐patient variability to exceed the heart displacement magnitude. Similarly, cardiac sparing remains patient‐specific in our study.

A major benefit of upright therapy, specifically for carbon ion therapy, is the increased flexibility of beam angles when using fixed beam lines. The same flexibility is achieved with the gantry, but currently, only four of the sixteen carbon‐ion facilities have access to a gantry. The necessity of a gantry has been investigated by Yan et al.^6^, ^7^ They demonstrated that a pencil beam scanning technique, eventually coupled with beam inflection and the sitting position could reduce the need for a gantry also for tumors with complex geometries. To investigate the dosimetric impact of the gantry for supine treatments, we optimized additional 3D supine plans only with vertical, horizontal and 45° fields, which are available in various combinations to all facilities treating thoracic lesions. Results show that for all patients, expect for P1, a full beam angle flexibility permits to achieve an increased plan quality (see Table S4). Despite possible dosimetric advantages, economical and practicality arguments are to be considered. For example, to treat a patient lying on the couch with a posterior beam without a gantry implies the patient has to be repositioned in prone position with the consequent acquisition of a prone CT. Thus, not only the treatment time, but also the sources of uncertainty and error would increase.

To properly investigate upright radiotherapy, it is necessary to compare it with the current clinical practice. Because of the shortage of current available gantries in clinical facilities, a more clinically relevant and realistic comparison can be performed between the fixed beam supine plans and the upright plans (4th column of Table S4 and 5th column of Table 2, respectively). The results show that with fixed beam supine plans a lower dose is delivered to the heart for patients P3, P4 and to the lung for patient P6. If a dosimetric equivalence between the two postures can be demonstrated, it would provide a strong argument to leverage the other opportunities offered by upright patient positioning. Not least, also the comfort for patients having trouble breathing in supine position^17^, ^36^ would benefit from upright positioning as an option. The renewed interest in upright therapy was mainly driven by economic arguments, and more specifically the possibility to replace the gantry with the patient rotation.

### 4.3 Plan robustness

When the patient immobilization is changed from recumbent to sitting on a chair, the whole treatment paradigm changes and main aspects of the workflow have to be revised, such as the magnitude of systematic errors. In fact, the margin recipe^18^ currently in use for radiotherapy treatments, was developed on a supine patient population and might not be applicable for upright treatment, because of inter‐fraction motion variation^37^ and position reproducibility.^18^ Therefore, we not only performed an RA for conventional robustness parameters of 3 mm/3.5%, but also for increased uncertainty scenarios, namely 5 mm setup position uncertainties and 5% density shift. Only a single scenario out of nine failed in two upright patients, while for the others, all scenarios passed the *D*_95%_/*V*_95%_ > 95% criteria, which was also the same passing rate as for supine plans. This indicates that the optimized plans would remain clinically valid also in the case of increased uncertainty in the upright position.

### 4.4 Interplay effect magnitude

Scanned carbon ion therapy with its sharp beam spots is particularly vulnerable to the interplay effect.^38^ A posture dependence of its dosimetric impact is therefore of interest. Previous studies, Duisters et al.^36^ and Yang et al.,^16^ suggested a reduced breathing amplitude in the upright position, which might also lead to a reduced interplay effect. The dataset used in this study is characterized by a general small motion amplitude, with a maximum of 6 mm for patient P2, the only case with the target in the lower lobe, where the maximum motion amplitude occurs.^39^ Indeed, P2 is the only patient for which the target coverage differs significantly between the two treatment positions, when the interplay effect is taken into account. Although for some of the other patients’ significant differences were observed, the magnitudes were clinically irrelevant, both in single fractions as well as the accumulated therapy course. This suggests that motion effects might be similar between postures, which could also extend to typical motion mitigation strategies. Since special motion management techniques are usually considered only for motion amplitudes exceeding 5 mm,^37^, ^40^ no optimization method to manage motion, that is, 4D techniques, was tested in this work. Indeed, target coverage for all patients except for P2 in supine, where the 5 mm threshold is violated, was adequate even for single fractions.

Both upright and supine V16Gy(lung) were lower than 30%, which is considered the upper threshold for a clinically accepted plan.^25^ Notably, upright plans result in an increased lung V16Gy(lung) both in % and in cc for all patients except P1.

It should be noted that the observed increase in the total lung volume indicates an increased air intake in upright posture, such that of the absolute volume in upright, a larger part consists of air rather than tissue.^13^

### 4.5 General comments and limitations

The investigation and assessment of each of the described variable allows to perform a thorough comparison between the two treatment modalities. Our results highlight the impact of each variable on the final dose comparison. Position‐specific angle optimization, different angle flexibility scenarios, as well as the choice of the contoured or propagated target, are main factors influencing the final plan comparison. However, our study demonstrates how patient‐specific characteristics can outweigh the effect of any of these variables. Our study underlines the importance of careful treatment selection based on patient‐specific lung anatomy and motion amplitude changes, as well as heart displacement. In particular, the upright position did not necessarily yield a lower V16Gy(lung), and the effect on the lung tissue requires additional attention. Nevertheless, we showed how different aspects of a treatment planning study, that is, plan robustness and convergence are not affected by the change in patient posture.

The main limitation of this work is the small patient cohort of six patients. This reflects the general lack of paired upright and supine 4DCTs available worldwide. Our results suggest no substantial differences between postures in 5 out of 6 patients, with selective benefits favoring either posture resulting from patient‐specific characteristics, such as the location of the tumor in the lungs. Moreover, the framework we developed highlights how the preference between upright and supine treatment strongly depends on the specific parameter settings underlying the comparison, emphasizing the importance of patient‐specific considerations. Differences observed under one comparison condition (e.g., 3D plans on the reference CT) did not translate to other conditions where more complexity was included (e.g., D4D doses). This is not unexpected and highlights the need for rigor in future comparison studies.

A further limitation derives from DIR and target propagation. Ideally the ribcage ROI should enclose the same lung subvolumes in both positions, but it was not possible to identify the exact same lung district in paired CTs. The optimal ribcage ROI definition is challenged by multiple factors, such as the anatomical changes, a different CT quality and the necessity to find a good trade‐off between small ribcage ROI to exclude body regions with large anatomical changes and big ribcage ROI to guarantee good anatomical match between supine and upright postures. In addition, a ground truth for the target contours in both postures was missing in our study, as the quality of the manual target contours differed between the CTs depending on whether the respective posture was actually used for therapy. Therefore, the registration and propagation QA were limited to the lung VOI, which is relatively straightforward to segment and register because of the high‐density contrast with the surrounding tissues. Since the vector fields are only constructed inside the ribcage ROI, the DIR QA was also restricted to this volume. Even if other structures, such as the heart, the spine and the rib cage were present in the selected ribcage ROI, only the heart was available in the original dataset for each patient, and depending on the tumor position in the lung, this could be outside the ribcage ROI. Still, the high quality in the propagation of the lung structure together with the good agreement in the target contour volumes, COM coordinates distance as well as qualitative shape between postures suggests that the ribcage ROI‐restricted DIR performed reasonably well.

One additional limitation was that the thorax chair at NMPC has a 20° backrest inclination, which should be reproduced in the TPS. However, this was not included in the optimization process, and for plan optimization and dose calculation a chair with vertical backrest was assumed, as the specific geometry of the chair installed at the NMPC is not included in our TPS. As the ideal patient posture is not yet clear, this additional parameter is also one that possibly influences a comparison between postures. It is important to point out that different vendors offer a variety of beam geometries and conditions possibly entailing vendor‐specific conclusions on the relative comparison between postures. It might be worth mentioning that a chair inclination other than vertical results in strictly non‐coplanar beams, and especially in the context of a treatment study, the beam travels across the typically inferior resolution of the slice thickness. This might have an impact on the accuracy of range estimates, which was avoided in this study by choosing planar beams.

## 5 CONCLUSIONS

This is the first work investigating the impact of upright patient positioning on carbon ion therapy for thoracic patients through a 4D dosimetric study, highlighting the complexity of the comparison with the standard supine treatments, due to the multiple variables playing a role. Breathing and beam motion, anatomical changes, plan robustness, beam angle direction, target geometry, target coverage as well as dose to the OARs are all factors to be taken into consideration. Our results seem to indicate that no evident upright and supine plan quality difference exists, and the choice of either treatment position has to be driven by patient‐specific characteristics. While a larger comparative effort is required as soon as more paired 4DCT data becomes available, our work demonstrates the sensitivity of the comparison to multiple variables and presents a comprehensive way to rigorously conduct such a study in the future. Indeed, to treat the patient in upright position, requires redesigning multiple treatment aspects, investigating and comparing the above‐mentioned variables with the current supine therapy, as done in this study.

## CONFLICT OF INTEREST STATEMENT

The authors declare no conflicts of interest.

## Supporting information




Supporting Information
