# Supplementary material for: Upright and supine particle therapy of lung cancer: A 4D dosimetric comparison
Source: Med Phys. 2026 Mar 3;53(3):e70377. doi: 10.1002/mp.70377 (PMC12957711; doi:10.1002/mp.70377)
Supplement: Supplementary file 1 — Supporting Information [file MP-53-0-s001.docx]

**Supplementary**

*Supplementary 1: Deformable image registration and target propagation*

We constrained DIR to a ribcage ROI, masking the CT in the reference breathing phase (end-inhale phase) for each supine and upright 4DCT around ribcage and the lung defining a ribcage ROI enclosing the ITV together with some high contrast structures. To maintain a minimum distance between ribcage ROI boundaries and the target contour, several slices (ranging from 3 to 25) were added to the ribcage ROI from the ITV superior and inferior limit. In the attempt of including the same anatomical regions in paired ribcage ROIs, a similar (not the same because of the different upright and supine CT slice distance) number of slices were added in cranio-caudal (CC) direction for the same patient in the two positions. This number varies between patients, depending on the tumor position and the anatomical changes magnitude between postures. In this way, we achieved similar paired ribcage ROI, while excluding body regions that highly differ between the upright and supine CT of the same patient, and that are responsible of a poor registration quality.

The DIR was performed with Plastimatch [42] in both directions (from the supine posture to the seated one and vice versa) on the ribcage ROI, and the vector fields were used to propagate the ITV. The Plastimatch algorithm used for DIR generation was based on three bspline registration stages, with regularization coefficient set to 0.1, and the mean standard error (MSE) as cost function metric to be optimized. No rigid registration was performed priorly to the DIR.

Registration and target propagation quality analysis (QA) were performed in 3DSlicer comparing ITV shape, dimension, and position in the thorax for a qualitative analysis. The target positions were visually compared in 3DSlicer, and the distance between ITV center of mass (COM) coordinates was also calculated for a more quantitative analysis. The COMs were extracted in 3D Slicer, with their coordinates computed relative to the supine image origin and to the supine origin propagated to the upright CT for the contoured and propagated ITVs, respectively. The premise of this analysis is to eliminate bias stemming from target contouring differences in both positions, no reliable ground truth was available for the tumor volume. To analyse the DIR in the ribcage ROI, dice similarity coefficient (DSC) and the average Hausdorff distance (AHD) metrics [28] were computed for the lung volume of interest (VOI) (only the subvolume enclosed in the ribcage ROI). The DSC, also known as overlap index, quantifies the similarity of superimposed segments, which would be equal to 1 for identical structures. The AHD, instead, is a distance metric based on the spatial position and dimension of voxels, and a good structure propagation should return a value smaller than the CT slice distance [29].

The DIR and propagation QA results are listed in Table S-1. Propagated and contoured lung VOIs comparison results in an average DSC and AHD values of 0.95 and 1.5 mm respectively.

The target propagation quality can only be assessed comparing structure shapes, positions and volumes. Upright and supine contoured ITV propagated ITVs, in both propagation direction, are listed in Table S-2. An average absolute volume difference of 5.7%±3.8% is obtained for all six patients and both propagation directions, with a maximum of 12.5% difference for upright ITV propagated on the supine CT for patient P6, and an exact equal volume for patient P5. Qualitatively, the volume shapes also remain consistent between postures as shown in Figure S-1. The COM coordinates distance between contoured ITV on supine CT and propagated ITV on upright CT are shown for all patients, except for P2 (same as Figure S-1). The largest difference was found for P1, while patients from P2 to P6 show COM distances of few mm (namely few voxels). The differences between COM coordinates derive both from position inaccuracies and contour deformation. Figure S-1 and Table S-3 refer to the targets used to optimize supine and upright nominal 3D plans.

| **Propagation**  **Direction** | **Patient** | **Contoured vs propagated VOI** | |
| --- | --- | --- | --- |
|  |  | **DSC** | **AHD (mm)** |
| **Supine 🡪 Upright** | P1 | 0.95 | 1.45 |
|  | P2 | 0.93 | 2.00 |
|  | P3 | 0.95 | 1.48 |
|  | P4 | 0.96 | 1.46 |
|  | P5 | 0.94 | 1.50 |
|  | P6 | 0.96 | 1.20 |
| **Upright 🡪Supine** | P1 | 0.93 | 1.50 |
|  | P2 | 0.96 | 1.52 |
|  | P3 | 0.96 | 1.36 |
|  | P4 | 0.95 | 1.42 |
|  | P5 | 0.92 | 2.00 |
|  | P6 | 0.96 | 1.24 |

Table S-1. DIR and lung VOI propagation QA. For each patient and each propagation direction the DSC and AHD values are listed.

| **Patient** | **Target volume (cc)**  **(Supine🡪Upright)** | | **Target volume (cc)**  **(Upright🡪Supine)** | |
| --- | --- | --- | --- | --- |
|  | **Supine** | **Upright** | **Supine** | **Upright** |
| **P1** | 60 | 58 | 68 | 65 |
| **P2** | - | - | 21 | 20 |
| **P3** | 381 | 364 | 361 | 340 |
| **P4** | 60 | 56 | - | - |
| **P5** | 273 | 262 | 244 | 244 |
| **P6** | 223 | 249 | 223 | 195 |

Table S-2. Manually contoured and propagated target volumes in both patient positions and for both propagation directions. For patients P2 and P4 the target was only contoured on the upright and supine CT, respectively.


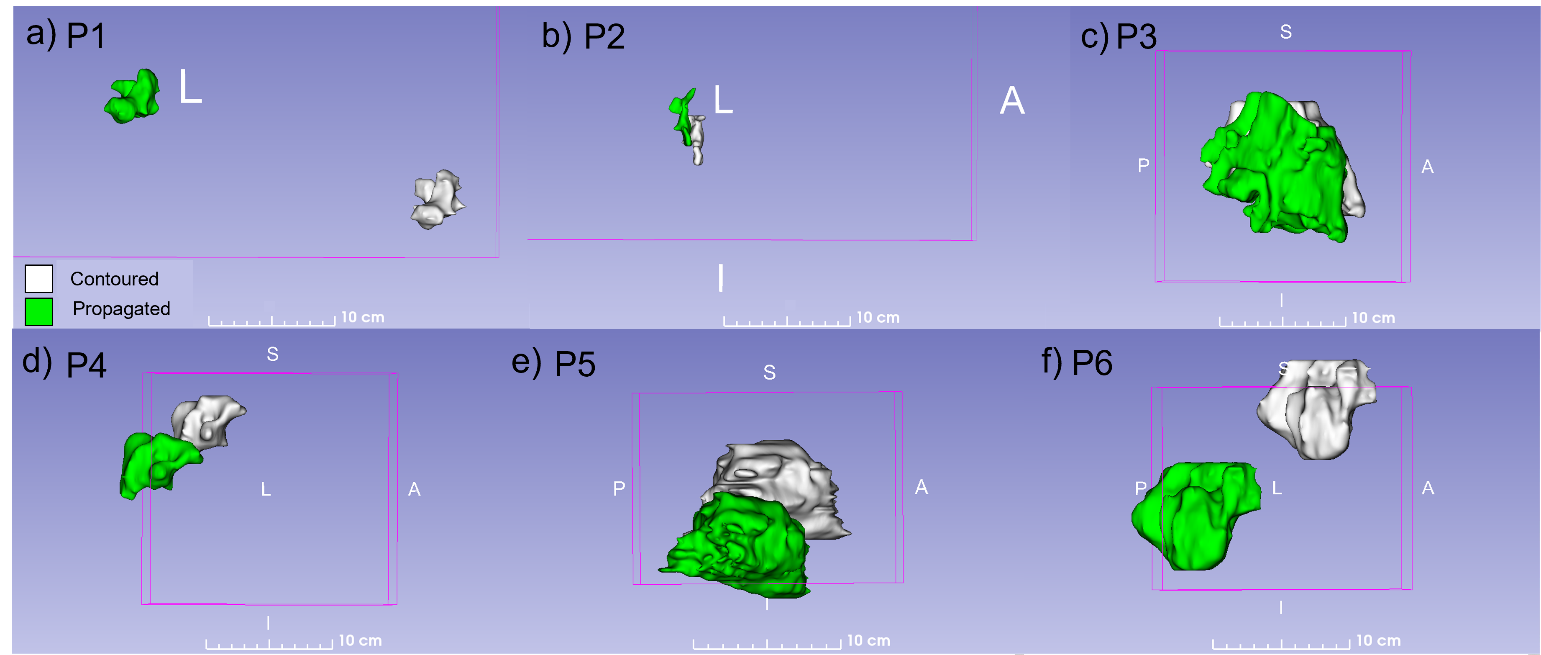


Figure S-1. Patient P1-P6 (a-f) original (white) and propagated (green) target in 3D view. Contoured supine target and the propagated one on the upright CT are shown. For patient P2, the contoured upright and propagated supine targets are shown. S=superior, I=inferior, A=anterior, P=posterior, L=left, R=right

| **Patient** | **Target ΔCOM (mm)**  **(LR, AP, IS)** |
| --- | --- |
| **P1** | 13.8, -57, -33.1 |
| **P2** | 1, 5.9, -22.4 |
| **P3** | -0.4, 12.1, -0.9 |
| **P4** | -4.9, 1, -9.2 |
| **P5** | -2.1, 8.8, -7.9 |
| **P6** | 1.4, -2.8, -6.7 |

Table S-3. Center of Mass distance (ΔCOM) between contoured ITV on supine CT and propagated ITV to the upright CT (Figure S-1). Distance vector components in left-right (LR), anterior-posterior (AP) and inferior-superior (IS) are listed. For patient P2, the contoured upright and propagated supine ITV are shown.

Supplementary 2: Gantry and fixed beam plans in supine position

| **Patient** | **Field angles (degrees)** | **Supine (Gantry)** | | **Field**  **angles**  **(degrees)** | **Supine**  **(Fixed Beamline)** | |
| --- | --- | --- | --- | --- | --- | --- |
|  |  | **V16Gy (%)** | **V20Gy (%)** |  | **V16Gy(%)** | **V20Gy(%)** |
| P1 | 90,290 | 10.4 | 0 | 90,270 | 9.7 | 0 |
| P2 | 250,290 | 9.4 | 5.5 | 225,315 | 12.4 | 7.2 |
| P3 | 90,270 | 23.0 | 15.0 | 90,270 | 23.0 | 15.0 |
| P4 | 270,290 | 4.3 | 2.8 | 270,315 | 7.5 | 3.1 |
| P5 | 270,135 | 17.3 | 8.4 | 270,135 | 17.3 | 8.4 |
| P6 | 250,290 | 19.9 | 5.0 | 225,315 | 28.5 | 5.1 |

Table S-4. Supine plans optimized mimicking the full beam direction availability of the gantry (1^st^-2^nd^ columns) and a fixed beamline scenario (3^rd^-4^th^ columns). Plans are compared through V16Gy(lung) and V20Gy(heart) for lung and heart dose, respectively. For patient P3 and P5 the selected angles from the gantry would have also been deliverable with a fixed beam, therefore these plans were not recalculated. Vertical AP 90 degrees, vertical PA 270 degrees, oblique AP 45 and 135 degrees, oblique PA 225 and 315 degrees. Field angles refer to the patient coordinate system and not the room system that would be different for the gantry and the chair.

Supplementary 3: Robustness Analysis


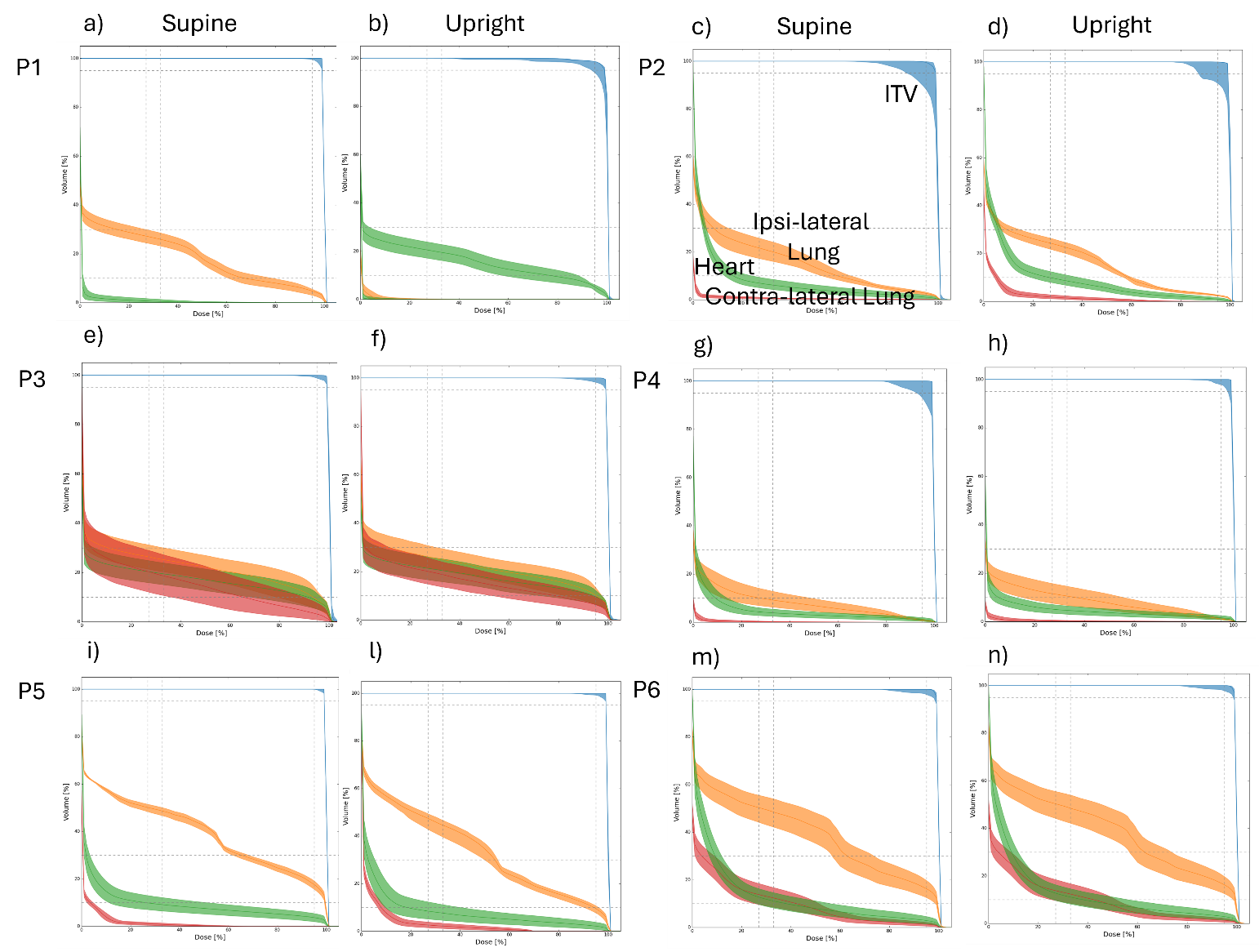


Figure S-2. Patients P1-P6 (a-n) supine (a, c, e, g, i, m) and upright (b, d, f, h, l, n) 5mm/5% robustness analysis DVHs. Ipsilateral lung (orange), contralateral lung (red), heart (green) and ITV (blue) nominal scenarios are plotted with solid lines, and bands illustrates the minimum-maximum range. The minimum clinically acceptable target coverage (D_95%=_95% and/or V_95%_= 95%), lung (V27%(lung)=30%) and heart dose (V33%(heart)=10%) are marked with dashed lines. ITV = internal target volume.

Supplementary 4: 4D delivered dose analysis

| **Patient** | **D_95_(%)**  **(µ±σ)** | | **HI (%)**  **(µ±σ)** | | **V_95_(%)**  **(µ±σ)** | |
| --- | --- | --- | --- | --- | --- | --- |
|  | **Supine** | **Upright** | **Supine** | **Upright** | **Supine** | **Upright** |
| **P1** | **96.1±0.2*** | 94.3±0.5 | **7.8±0.5*** | 11.2±0.8 | **98.2±0.5*** | 92.9±1.8 |
| **P2** | 91.7±0.8 | **94.6±0.5*** | 16.2±1.2 | **11.0±0.7*** | 84.4±3.4 | **93.9±1.7*** |
| **P3** | 97.3±0.1 | 97.3±0.1 | 5.6±0.1 | **5.5±0.1** | 99.6±0.1 | **99.7±0.1** |
| **P4** | 94.9±0.4 | **95.0±0.5** | 9.9±0.7 | 9.9±0.4 | 95.0±1.1 | 95.0±1.4 |
| **P5** | **97.5±0.1*** | 97.3±0.2 | **5.0±0.2*** | 5.4±0.4 | **99.8±0.1*** | 99.3±0.3 |
| **P6** | 95.4±0.2 | **95.7±0.2*** | 9.1±0.4 | **8.5±0.2*** | 96.2±0.6 | **97.2±0.5*** |
| **All** | **95.8±0.01** | 95.5±0.01 | **8.3±0.1** | 8.9±0.2 | 95.5±0.2 | **96.7±0.1** |

Table S-5. Upright and supine D_95%_, HI and V_95%_ metrics averaged over 20 deliveries. Mean (µ) and standard deviation (σ) are listed. Statistically significant better values (p<0.05) are marked with an asterisk and are highlighted in bold. In the last row, the average over the six patients are listed.

| **Patient** | **V16Gy (Lung) (%)**  **(µ±σ)** | | **V16Gy (Lung) (cc)**  **(µ±σ)** | | **V20Gy (Heart) (%)**  **(µ±σ)** | |
| --- | --- | --- | --- | --- | --- | --- |
|  | Supine | Upright | Supine | Upright | Supine | Upright |
| **P1** | 10.8±0.03 | **9.6±0.1** | 245.0±0.6 | **227.5±2.8** | 0 | 0 |
| **P2** | **9.6±0.05** | 11.5±0.09 | **377.7±1.9** | 527.4±4.1 | 6.0±0.06 | **8.6±0.2** |
| **P3** | **23.2±0.02** | 23.7±0.02 | **785.4±0.6** | 938.8±0.9 | **18.6±0.1** | 18.8±0.06 |
| **P4** | **3.8±0.07** | 4.3±0.1 | **124.9±2.3** | 150.3±2.5 | **3.8±0.04** | 4.6±0.02 |
| **P5** | **17.8±0.03** | 19.5±0.06 | **469.6±0.8** | 618.5±1.8 | 9.3±0.02 | **7.5±0.02** |
| **P6** | **20.4±0.1** | 25.6±0.1 | **663.6±3.5** | 797.1±3.8 | **6.1±0.09** | 11.0±0.08 |
| **All** | **14.3±0** | 15.7±0 | **444.4±0.2** | 543.2±0.3 | **7.3±0** | 8.4±0 |

Table S-6. Upright and supine lung (V16Gy(lung)) and heart (V20Gy(heart)) dose metric. V16Gy(lung) is shown both in percentage and volume. Values are averaged over 20 deliveries, mean (µ) and standard deviation (σ) are listed. For each comparison, the better value (non-significant) is highlighted in bold. In the last row, the average over the six patients are listed.


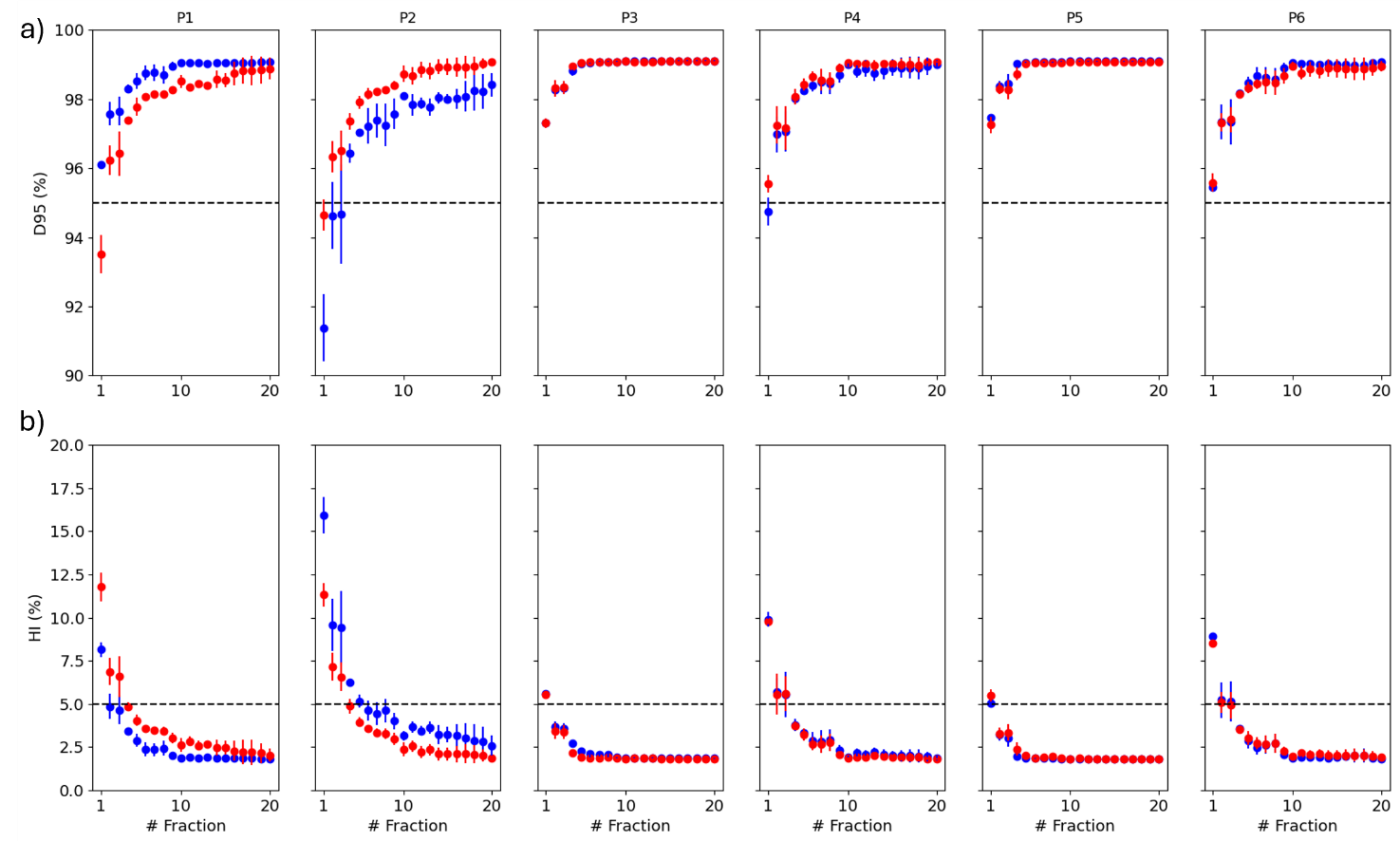


Figure S-3. Patients P1-P6 target coverage convergence over 20 fractions. D_95%_ and HI are shown both for upright (red) and supine (blue) plan. Four fractionation treatments are simulated, and their mean (dot) and standard deviation (bars) are displayed for each fraction. The minimum clinically acceptable values of 95% and 5% are marked with dashed lines for D_95%_ and HI, respectively.
